# Multidimensional Evaluation of Fruit Color Differentiation in *Rubus hirsutus* Thunb.: Significant Differences in Morphological, Nutritional, and Physiological Characteristics Between Red and Yellow Berry Varieties

**DOI:** 10.1155/ijfo/8921700

**Published:** 2026-04-24

**Authors:** Xiaobao Tong, Xingru Wei, Qilong Zeng, Xinyue Ping, Liangqin Liu, Yanqin Jiang, Xiaomin Wang

**Affiliations:** ^1^ Jiangsu Key Laboratory for Conservation and Utilization of Plant Resources, Institute of Botany, Jiangsu Province and Chinese Academy of Sciences (Nanjing Botanical Garden Memorial Sun Yat-Sen), Nanjing, 210014, Jiangsu, China, botanica.sp.gov.br

**Keywords:** antioxidant indicators, fruits, germplasm resources, nutritional components, *Rubus hirsutus* Thunb.

## Abstract

*Rubus hirsutus* Thunb., a typically red‐fruited shrub in the Rosaceae family, was found to include a stable yellow‐fruited variety at the Zhongshan Botanical Garden in Nanjing, China. This study provides the first comprehensive and multidimensional comparison of red (R) and yellow (Y) *R. hirsutus* fruits across three developmental stages (immature, color‐turning, and mature), integrating morphological, sensory, nutritional, and antioxidant analyses. For the first time, we systematically characterized sugar composition, amino acid profiles, phenolic compounds, and multiple antioxidant parameters (including total phenols, anthocyanins, glutathione, and antioxidant enzymes) in both color variants. Results revealed that mature yellow fruits exhibited significantly larger size (diameter and weight) than red fruits. While red fruits showed brighter coloration, higher soluble solid–acid ratio, and superior flavor scores, they accumulated significantly more fructose, total phenols, anthocyanins, trace elements (Fe and Zn), glutathione, and peroxidase activity at maturity. Conversely, yellow fruits contained higher levels of titratable acid (0.89% vs. 0.73%), total amino acids (2263.52 vs. 1835.68 μg/g in red), specific essential amino acids (His, Trp, and Ile), potassium, and a higher K/Na ratio. The protein content did not differ significantly between the phenotypes. Correlation analysis revealed that morphological indices were closely associated with sugar accumulation, whereas antioxidant indicators showed limited correlation with other parameters. These novel findings not only fill a critical gap in the characterization of yellow‐fruited Rubus germplasm but also provide a scientific foundation for targeted breeding and functional food development.

## 1. Introduction

Encompassing roughly 700 species, the genus *Rubus* L. (Rosaceae) stands as a highly diverse group of plants, primarily composed of deciduous shrubs alongside fewer evergreen shrubs or perennial herbs. The species exhibits a broad distributional range, primarily across the temperate and tropical regions of the Northern Hemisphere, with scattered populations in the Southern Hemisphere [1–3]. Characteristic morphological features include stems with prickles or bristles and aggregate fruits composed of small drupelets [2]. Abundant wild germplasm of Rubus is found in China, one of its centers of genetic resources, with its primary distribution concentrated in the southwestern region and extending to the eastern (Jiangsu, Anhui, Zhejiang, Jiangxi, and Fujian) and southern (Guangxi) parts of the country [4, 5]. *Rubus hirsutus* Thunb. is a representative native species of the Rubus genus in the Jiangnan region of China, valued for its ecological adaptability and development potential. The plant exhibits strong upright growth, produces pure white flowers up to 4–5 cm in diameter during its full blooming period in March, and bears subglobose fruits (approx. 2 cm in diameter) that ripen to a bright red color typically in April–May [6, 7]. Owing to its compact growth form, notable cold tolerance, ease of propagation, and low maintenance needs, *R. hirsutus* possesses considerable horticultural merits, positioning it as a high‐quality candidate for breeding edible raspberry cultivars in southern China [6].

Beyond being appealing sensory qualities, *R. hirsutus* fruits are dense in a variety of nutrients, such as organic acids, minerals, and components known for their significant bioactivity. Notably, the superoxide dismutase (SOD) activity in its fruits is remarkably high among edible raspberries in southeastern China [4], reaching up to 388 U/g, significantly exceeding that of most comparable varieties. The fruits are abundant in secondary metabolites, which are key to their purported medicinal functions (e.g., antioxidant, anti‐inflammatory capacities, and hepatoprotective) [8] and their potential as raw materials for functional foods/nutraceuticals [3, 4, 6, 8, 9]. Berry fruits, with *R. hirsutus* as an example, serve as concentrated sources of essential nutrients and diverse bioactives. Their dietary inclusion can support human health by mitigating the development risk of numerous chronic diseases and metabolic syndromes [10]. A wide range of constituents underlies the nutritional and functional benefits offered by these fruits. Key components comprise vitamins, minerals, and dietary fiber, alongside important bioactive polyphenols, such as anthocyanins, flavonoids, and phenolic acids, in addition to various amino and fatty acids [10–12]. These compounds collectively underpin the distinctive flavor, aroma, and health‐promoting properties of berries. Their benefits include potent antioxidant activity that scavenges free radicals and combats oxidative stress, potentially delaying the aging process and reducing the incidence of age‐related pathologies, notably cardiovascular and neurodegenerative diseases [8, 11, 13]. Furthermore, a range of other bioactivities have been reported for *Rubus* berries, including potential anti‐inflammatory, anti‐ulcer, antitumor, anti‐obesity, hypoglycemic, immunomodulatory, and antimicrobial effects [8]. Consequently, *R. hirsutus* and other *Rubus* berries possess not only basic nutritional value but also the attributes of “functional foods” due to their rich bioactive compounds, offering specific health benefits alongside meeting nutritional needs [10]. Their high genetic diversity also makes them important scientific research material. Furthermore, secondary metabolites and fiber components from various parts of *Rubus* plants, including *R. hirsutus* (e.g., fruits and bark), hold potential applications in cosmetics and the fiber industry [3, 6]. Based on their ethnopharmacological background (e.g., used to alleviate specific gynecological symptoms [14]) and modern pharmacological research, the development prospects for *R. hirsutus* and its products (e.g., juice [15], dietary supplements, and pharmaceutical preparations) are promising.

Despite the extremely rich germplasm resources of the genus *Rubus* L. in China, harboring numerous accessions with potential application value, most remain wild or semiwild with low utilization [16]. Due to the complex structures of their active components (especially secondary metabolites), systematic phytochemical studies on *Rubus* plants in China are still insufficient [17]. Although Saini et al. reported on the antioxidant, antimicrobial, and antiproliferative activities of some *Rubus* species [18], an in‐depth chemical characterization of key active constituents, such as phenolic compounds, as well as other chemical profiles for specific germplasms or types, is still lacking. Globally, cultivated raspberries (*R.* spp.) have developed various fruit color types, including black, purple, and gold, but red‐fruited cultivars are the most common [19]. Notably, this study identified a yellow‐fruited germplasm of *R. hirsutus* Thunb. Current research on *R. hirsutus* primarily centers on two aspects: analyzing chemical composition disparities in fruits of varying sizes [20] and examining the dynamic changes in physicochemical properties, phytochemical composition and associated antioxidant activity, and mineral content across immature, semimature, and fully mature stages [21]. Despite these efforts, a systematic comparison focusing specifically on fruit color variation—particularly between the common red‐fruited type and the rare yellow‐fruited germplasm—remains absent. In particular, little is known about how color differentiation relates to key nutritional components, bioactive compounds, and functional attributes, such as antioxidant capacity. To address this gap, this study provides the first integrated multidimensional evaluation of red (R) and yellow (Y) fruits of *R*. *hirsutus*. In a novel approach that extends beyond previous single‐parameter studies, mature fruits of both color variants were systematically analyzed for sugar composition, phenolic profiles (with an emphasis on previously rarely characterized characteristic phenolics), comprehensive amino acid patterns, and multiple dimensions of antioxidant capacity (including free radical scavenging, total antioxidant activity, and individual antioxidant components, such as glutathione (GSH) and anthocyanins). This represents the first systematic biochemical and functional characterization of the yellow‐fruited *R. hirsutus* variety, revealing previously unknown metabolic signatures associated with fruit color variation. This work not only fills a critical void in the systematic chemical and functional assessment of yellow‐fruited Rubus germplasm but also offers essential data for the accurate identification, evaluation, and differentiation of the nutritional and functional values of red and yellow *R. hirsutus* accessions. Furthermore, these findings provide a foundational basis for future efforts in targeted breeding, functional food development, and the enhanced utilization of this valuable yellow‐fruited resource.

## 2. Materials and Methods

### 2.1. Experimental Materials

The plant cultivation for this study was conducted at the Nanjing Botanical Garden Mem. Sun Yat‐Sen (longitude 118°49′E, latitude 32°3′N), which hosted the red‐ (R) and yellow‐fruited (Y) accessions of *R. hirsutus*. Fresh wild fruits from both varieties at three developmental stages—immature green (S1), color‐turning (S2), and mature (S3)—were collected as experimental materials Figure 1. For each fruit variety (red and yellow) and each developmental stage (S1, S2, and S3), three independent biological sample groups were collected from different trees. Each biological replicate consisted of approximately 200–500 g of fruits. All samples were immediately frozen in liquid nitrogen upon collection and stored at −80°C until analysis.

FIGURE 1Fruits of two varieties of *R*. *hirsutus* at three developmental stages. Note: Depicted are the fruits of *R. hirsutus* at different developmental stages: (a) Immature red; (b) color‐turning red; (c) mature red; (d) immature yellow; (e) color‐turning yellow; (f) mature yellow. (bar = 0.5 cm).

**Figure:**
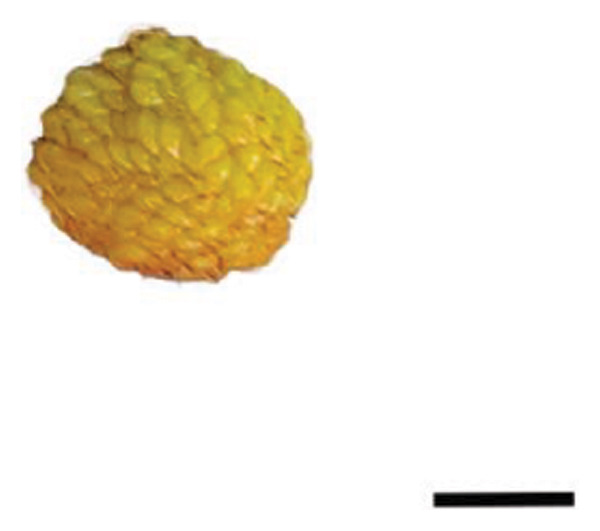
(a)

**Figure:**
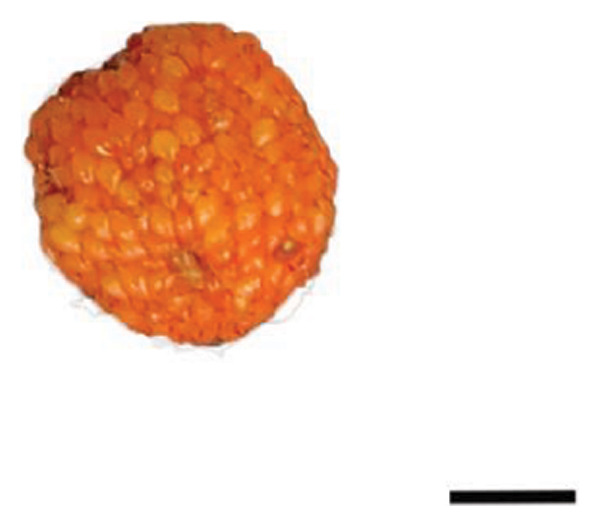
(b)

**Figure:**
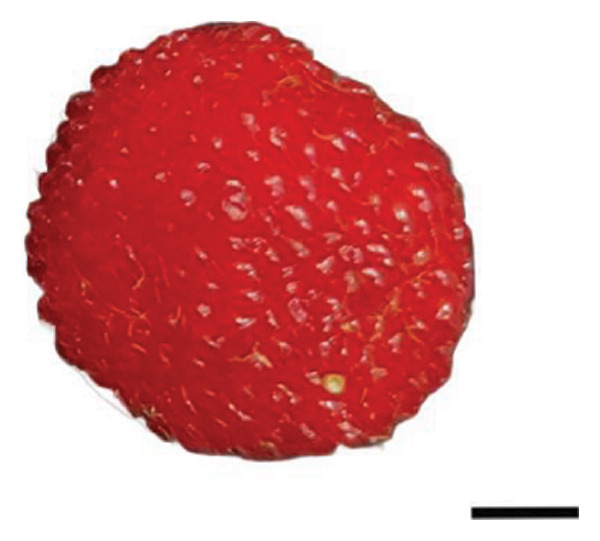
(c)

**Figure:**
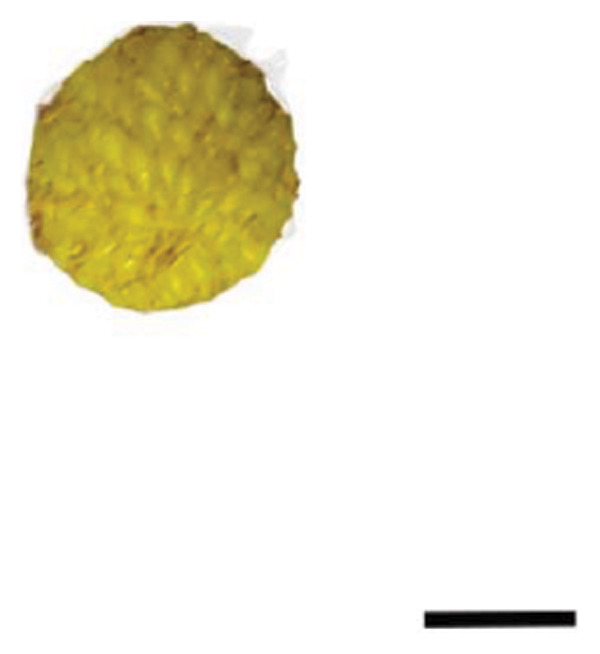
(d)

**Figure:**
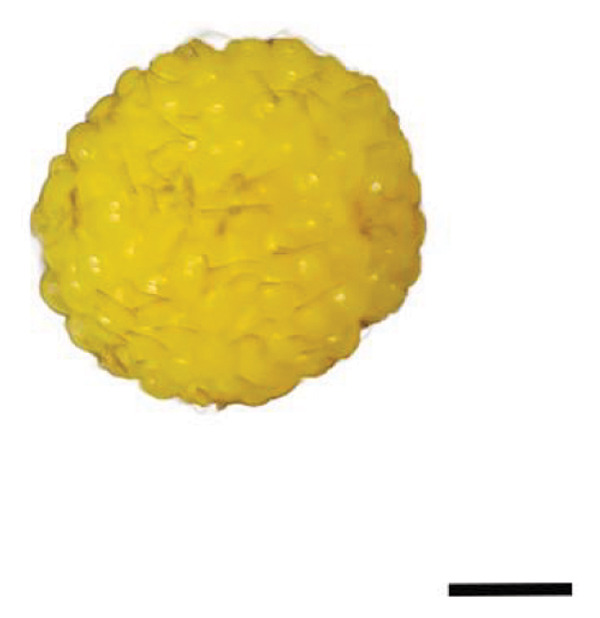
(e)

**Figure:**
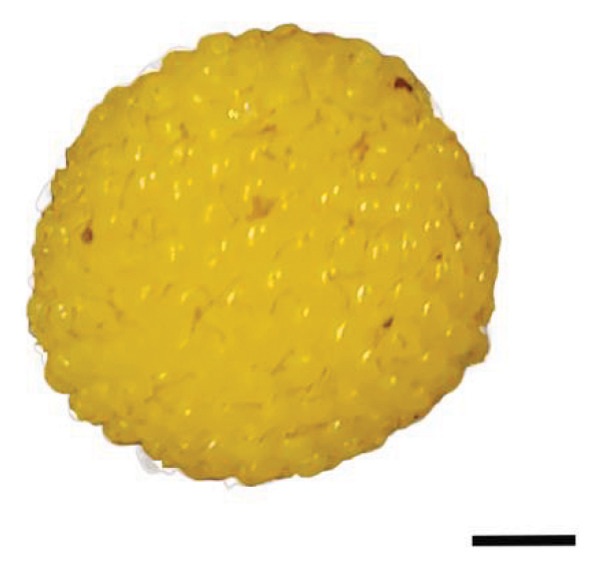
(f)

### 2.2. Main Reagents and Instruments

Reagents: Nitric acid (trace metal grade, Merck, Germany); acetonitrile (chromatographic grade, Merck, Germany); sodium acetate (analytical grade, Sinopharm, China); triethylamine (analytical grade, Sinopharm, China); Folin–Ciocalteu reagent (2N, Sigma‐Aldrich, USA); phosphate buffer solution (0.05 M, pH 7.8, prepared in‐house); and absolute ethanol (analytical grade, Sinopharm, China).

Standards: Fructose (HPLC grade, ≥ 99%, Sigma‐Aldrich, St. Louis, MO, USA), glucose (HPLC grade, ≥ 99%, Sigma‐Aldrich), and sucrose (HPLC grade, ≥ 99%, Sigma‐Aldrich) standards were used for sugar quantification. Amino acid mixed standard solution (Type H, 2.5 μmol/mL each) was purchased from Wako Pure Chemical Industries (Osaka, Japan).

Instruments*:* Microplate reader (Scientific Multiskan Sky, Thermo Fisher Scientific, China); electronic balances (Mettler LE204E, 0.0001 g precision; FA1204, Shanghai Hengji Scientific Instrument Co., Ltd., China); Agilent 7900 ICP‐MS; TG‐16G benchtop high‐speed centrifuge (Hunan Keda Scientific Instrument Co., Ltd.); Agilent 1260 liquid chromatography system coupled with an AB4000 mass spectrometer; PS40A ultrasonic cleaner (Shenhua Tai Scientific Instrument Co., Ltd.); and water bath nitrogen blower (Shanghai Naai Instrument Co., Ltd.).

### 2.3. Experimental Methods

#### 2.3.1. Determination of Fruit Morphological Characteristics

Fruit size was measured using a vernier caliper (Deli‐91150) to determine longitudinal and transverse diameters. Fruit mass was recorded with an electronic balance (0.001 g precision). Fruit color was measured on the fruit surface using an SR220 spectrophotometric colorimeter. Each parameter was measured on 10–15 fruits.

#### 2.3.2. Determination of Fruit Sensory Attributes

Sensory evaluations were conducted on fresh fruits at the maturity stage (S3) of both color morphs. The samples were placed in odorless transparent plastic cups and tested under white light at room temperature. Evaluators were allowed to rinse their mouths with distilled water between tasting different samples. For each plant, 2–3 fruits were tasted for the following evaluations.

Five attributes were evaluated using a 10‐point rating scale (1 = *extremely disliked/unacceptable*, and 10 = *extremely liked/intense*).

1. Appearance (color, uniformity, and plumpness) was scored based on preference (1–10).
2. Fruit aroma intensity was scored (1–10), with 1 being extremely faint and 10 being intense.
3. Sweetness and acidity intensity were scored (1–10).
4. Mouthfeel was scored based on preference (1–10).
5. A comprehensive score was given for each variety based on preference (1–10).

#### 2.3.3. Determination of Fruit Nutritional Component Contents

##### 2.3.3.1. Sugar Content

For sugar analysis, 0.5 g of samples representing different fruit colors and developmental stages were homogenized in a 4.5 mL volume of 0.05 M phosphate buffer (pH 7.8). Homogenates were then subjected to centrifugation, and the resulting supernatant was assessed for fructose, sucrose, and glucose concentrations with commercial assay kits (Jiancheng Bioengineering Institute, Nanjing, China) [22].

1. For fructose quantification, the supernatant was diluted 10‐fold and mixed with the substrate reaction solution. The mixture was incubated in a boiling water bath for precisely 8 min and then cooled under running water, before absorbance measurement of the reaction product at 285 nm.
2. Following the glucose oxidase method [23], glucose was quantified by adding the working solution to the supernatant; after gentle mixing, the reaction proceeded at 37°C for 10 min before absorbance reading at 505 nm.
3. Sucrose content was determined by the ultraviolet colorimetric method. The sample was thoroughly mixed with the hydrolysis solution, reacted in a 100°C water bath for 8 min, and cooled under running water, before absorbance quantification at 290 nm.

Blank tubes and standard tubes were set for all determinations, and each determination was performed in triplicate. Sugar concentrations were calculated based on the standard curves provided by the assay kits and expressed as mg/g fresh weight (FW).

##### 2.3.3.2. Titratable Acidity (TA)

TA was measured by potentiometric titration and calculated as citric acid using a conversion factor of 0.064. Fruit pulp (3 g) was homogenized with distilled water to a final volume of 30 mL and extracted at 65°C for 1 h. Following extraction, the sample was subjected to centrifugation at 4500 rpm for 10 min before titration. The resulting supernatant was then titrated with 0.1 mol/L NaOH to a pH endpoint of 8.0–8.1, and the TA content was calculated based on the volume of NaOH consumed.

##### 2.3.3.3. Soluble Solid Content (SSC)

The SSC was determined on representative samples of the fruit pulp using a PAL‐1 digital handheld refractometer. Before measurement, the fruits were thoroughly homogenized with a crusher.

##### 2.3.3.4. Protein Content

Total protein was quantified by the Bradford method [24] using a commercial assay kit (Nanjing Jiancheng Bioengineering Institute, Nanjing, China) according to the manufacturer’s protocol. Fresh tissue samples (0.5 g) were processed by homogenization in 4.5 mL of phosphate buffer (0.05 mol/L, pH 7.8) on ice and subsequent centrifugation at 8000 rpm for 10 min (4°C). A 0.1 mL aliquot of the supernatant was then mixed with 2 mL of Coomassie Brilliant Blue reagent. The mixture was incubated for 10 min at room temperature before measuring the absorbance at 595 nm. The protein content was expressed as mg/g FW.

##### 2.3.3.5. Amino Acid Content

The content of 31 amino acids was determined according to GB 5009.124‐2016 (National Food Safety Standard—Determination of Amino Acids in Foods) using an amino acid analyzer coupled with HPLC‐MS/MS technology. Following thawing, samples were processed by 10 min of ultrasonication, 2 min of vortex mixing, and filtration through a 0.22 μm aqueous phase membrane, with subsequent analysis conducted on an Agilent 1260 LC system coupled to a Xevo TQ‐S mass spectrometer.

##### 2.3.3.6. Plant Mineral Element Content

The concentrations of mineral elements were quantified following the protocol of the Chinese National Food Safety Standard (GB 5009.268‐2016) for the determination of multielements in foods. About 0.1 g of sample was digested with 5 mL HNO_3_ overnight in a polytetrafluoroethylene digestion vessel using a programmed temperature gradient (80°C for 2 h, 120°C for 2 h, 160°C for 4 h). The cooled digest was subjected to evaporation until near dryness, transferred quantitatively to a 25‐mL volumetric flask, and diluted to the mark with 1% HNO_3_, before instrumental analysis using a NexION® 1000 ICP‐MS. A reagent blank was prepared simultaneously.

#### 2.3.4. Determination of Antioxidant Substance Contents

##### 2.3.4.1. Total Phenolic Content

The Folin–Ciocalteu method was employed for total phenol quantification [25]. Sample preparation involved extracting 0.1 g of cryogenically powdered material with 2 mL of 60% aqueous ethanol, assisted by vortex mixing (2 min) and ultrasonication (60°C, 60 Hz, 3 min). After centrifugation (5000 rpm, 10 min), the supernatant was collected for analysis. For the color development reaction, 1 mL of the supernatant was sequentially combined with 0.5 mL of 0.2 mol/L Folin–Ciocalteu reagent and 2 mL of a 7.5% sodium carbonate solution. The mixture was then incubated in the dark at ambient temperature for 2 h, after which the absorbance was measured at 765 nm.

Total phenolic content was quantified using a commercial assay kit (Nanjing Jiancheng Bioengineering Institute, Nanjing, China) according to the manufacturer’s protocol. Results were expressed as mg gallic acid equivalents (GAE) per gram FW.

##### 2.3.4.2. Total Flavonoid Content

The total flavonoid content was determined using a colorimetric assay with aluminum chloride [26]. Powdered samples (0.05 g) underwent extraction with 2 mL of 60% ethanol at 60°C for 2 h with constant shaking. Following extraction and centrifugation (10,000 rpm, 10 min), the absorbance of the resulting supernatant was determined at 502 nm. The calibration curve was constructed using catechin standard at six concentrations (0, 10, 20, 30, 40, and 50 μg/mL) in 60% ethanol. The linear regression equation was *y* = 9.0771*x* + 0.0141 (*R* ^2^ = 0.9998), where y is the absorbance at 502 nm, and x is the catechin concentration (mg/mL). The concentration was calculated based on the standard curve. Results were expressed as mg catechin equivalents (CE) per gram FW.

##### 2.3.4.3. Anthocyanin Content

The anthocyanin content was determined using a pH differential method with UV–Vis spectroscopy [27]. A 3.0 g aliquot of pulp was homogenized and subjected to extraction with 30 mL of 50% ethanol. The mixture was then treated with ultrasound (35°C, 60 Hz) for 20 min, followed by centrifugation at 5000 rpm for 5 min. Subsequently, 300 μL of the resulting supernatant was blended with 2.7 mL of buffer solution adjusted to pH 1.0. This mixture was incubated in the dark for 15 min, after which its absorbance was determined at a wavelength of 510 nm with a blank solution as the reference. The calibration curve was constructed using cyanidin‐3‐O‐glucoside standard at five concentrations (0, 10, 20, 50, and 100 μg/mL) in pH 1.0 buffer. The linear regression equation was *y* = 0.0455*x* − 0.0881 (*R* ^2^ = 0.996), where y is the absorbance at 510 nm, and x is the anthocyanin concentration (μg/mL). Results were expressed as mg cyanidin‐3‐O‐glucoside equivalents per 100 g FW.

##### 2.3.4.4. GSH Content

The GSH content in *R. hirsutus* fruits was quantified using a commercial detection kit (Jiancheng Bioengineering Institute, Nanjing, China). Briefly, 0.5 g of fresh sample was homogenized in 4.5 mL of ice–cold phosphate buffer (0.1 M, pH 7.4) within an ice‐water bath. The resulting homogenate was kept at 4°C and subsequently centrifuged at 1000 rpm for 10 min to obtain the supernatant for analysis. Absorbance readings were taken at 420 nm and 450 nm according to the manufacturer’s protocol, and the GSH concentration was determined based on the kit’s standard curve and calculation formula. The GSH content was expressed as mg/g FW.

#### 2.3.5. Determination of Antioxidant Enzyme Activities (Peroxidase [POD] and SOD)

According to the manufacturer’s instructions (Jiancheng Bioengineering Institute, Nanjing, China), the activities of POD and SOD were quantified. Briefly, 0.5 g of fresh fruit tissue was homogenized in 4.5 mL of ice–cold phosphate buffer (0.1 mol/L, pH 7.4). Following centrifugation at 4°C and 1000 rpm for 10 min, the supernatant was analyzed, with absorbance measured at 420 nm for POD and 450 nm for SOD. The enzyme activities were expressed as U/g FW.

### 2.4. Data Processing and Analysis

All experiments were performed with three biological replicates, and each biological replicate was measured with three technical replicates. Data were expressed as mean ± standard deviation (SD). Each of the above indicators was measured in triplicate. The experimental data were organized and analyzed using Excel 2024.

Normality and Homogeneity of Variance: Normality of data distribution was assessed using the Shapiro–Wilk test, and homogeneity of variances was checked using Levene’s test. All data met the assumptions of normality and homogeneity of variance (*p* > 0.05), permitting the use of parametric tests.

Comparisons Between Red and Yellow Fruits at the Same Developmental Stage: Independent‐samples *t*‐tests were performed to compare red (R) and yellow (Y) fruits at each developmental stage (S1, S2, and S3) for all measured parameters (morphological traits, color indices, nutritional components, antioxidant substances, and enzyme activities).

Comparisons among Developmental Stages within the Same Fruit Color: One‐way analysis of variance (ANOVA) was used to evaluate differences across the three developmental stages (S1, S2, and S3) within each fruit color type (red or yellow). When significant differences were detected by ANOVA (*p* < 0.05), post hoc multiple comparisons were conducted using Fisher’s least significant difference (LSD) test. Results are presented with lowercase letters indicating significant differences (different letters denote *p* < 0.05).

**FIGURE 2:**
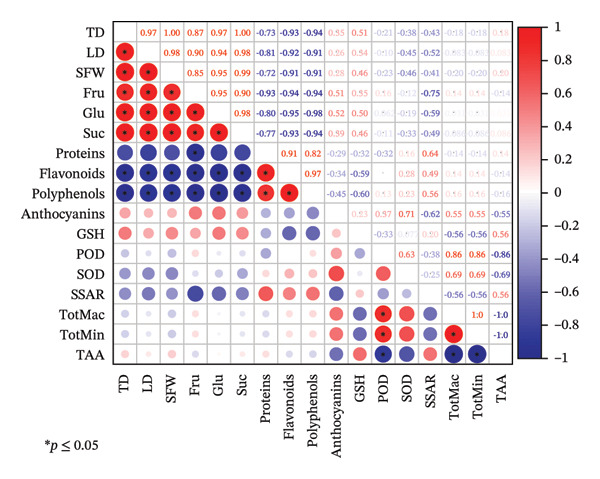
Correlation analysis between morphological, nutritional, and antioxidant parameters of *R. hirsutus*. Note: Pearson’s correlation coefficients were calculated. Color intensity represents the strength of correlation (red: positive, blue: negative, ^∗^ *p* ≤ 0.05).

Correlation Analysis: Pearson’s correlation coefficients were calculated to assess relationships between morphological indices, nutritional components, antioxidant parameters, and mineral elements. Correlation matrices were generated to visualize significant associations (Figure 2).

Note on Two‐Way ANOVA: Two‐way ANOVA to assess the interaction between fruit color and developmental stage was not performed, as the primary focus of this study was to compare the two color types at each stage separately and to examine developmental changes within each color type.

All statistical analyses were performed using SPSS Version 27 (IBM Corp., Armonk, NY, USA). Graphs were generated using Origin 2024 (OriginLab Corp., Northampton, MA, USA). A significance level of *p* < 0.05 was used for all tests.

## 3. Results and Analysis

### 3.1. Morphological Traits of *R. hirsutus* Fruits

To assess morphological distinctions between the two fruit color types, measurements were taken along the horizontal and vertical axes, along with single fruit mass. Figure 3 shows that yellow fruits had significantly larger transverse diameters and weights than red fruits across all three stages, especially at S3, where Y’s transverse diameter was 2.63 mm larger, and weight was approximately 0.6 g heavier than R’s. For longitudinal diameter, R was larger than Y at S1; the values intersected at S2; and Y remained larger than R at S3, exceeding R by 38.9%.

FIGURE 3Morphological characteristics of *R. hirsutus*. Note: Red (R) and yellow (Y) fruits of *R. hirsutus* are shown. Data are presented as mean ± SD (*n* = 3). Morphological parameters: (a) transverse diameter, (b) longitudinal diameter, and (c) single fruit weight.

**Figure:**
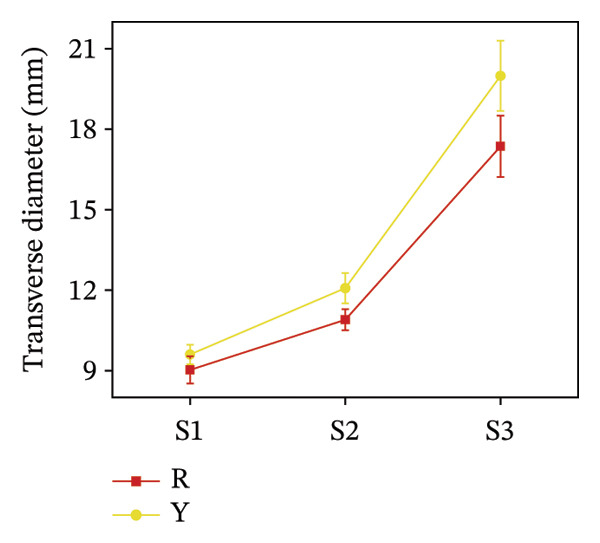
(a)

**Figure:**
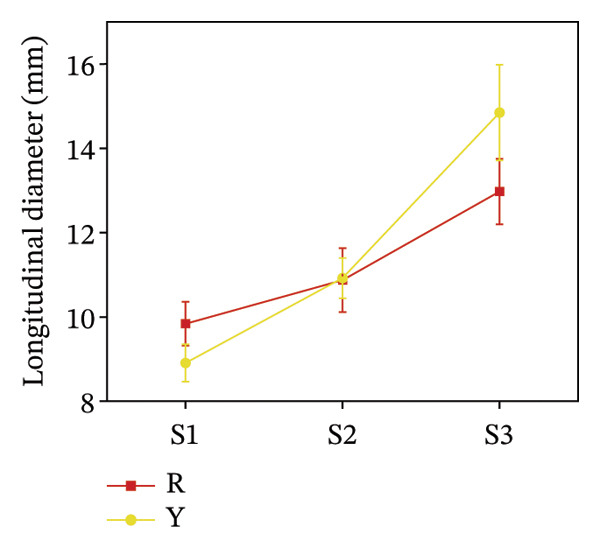
(b)

**Figure:**
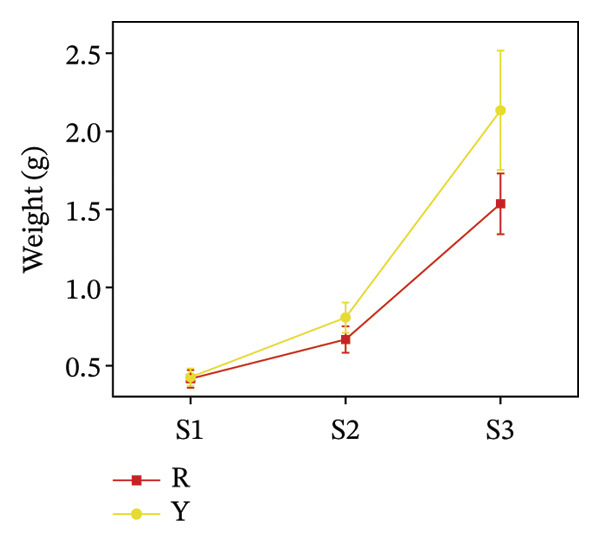
(c)

### 3.2. Color Difference Indicators of *R. hirsutus* Fruits

The average lightness (*L* ^∗^), average red–green value (*a* ^∗^), and average yellow–blue value (*b* ^∗^) of fruits are quantitative indicators of appearance quality and can reflect the ripeness of fruits [28]. Figure 4 illustrates the colorimetric changes, showing that the average lightness (*L* ^∗^) of red fruits reached its maximum (43.08 ± 2.6) at the immature S1 stage and gradually diminished as the fruits ripened. The average lightness of yellow *R. hirsutus* fruits peaked at the S2 stage (54.2 ± 4.5) and decreased after ripening. Color development during fruit maturation revealed distinct patterns: The *a* ^∗^ value of red fruits shifted from −5.79 ± 1.79 (dark green) to 24.19 ± 7.65 (bright red), whereas yellow fruits changed from −3.90 ± 1.45 (dark green) to 4.49 ± 1.52 (bright yellow). Meanwhile, the *b* ^∗^ value for both fruit types reached its maximum at the S2 stage, measuring 27.89 ± 10.17 for red and 36.1 ± 0.19 for yellow fruits.

FIGURE 4Color parameters of *R. hirsutus*. Note: Red (R) and yellow (Y) fruits of *R. hirsutus* are shown. Data are presented as mean ± SD (*n* = 3). Parameters shown: (a) average lightness (*L* ^∗^), (b) average red–green value (*a* ^∗^), and (c) average yellow–blue value (*b* ^∗^).

**Figure:**
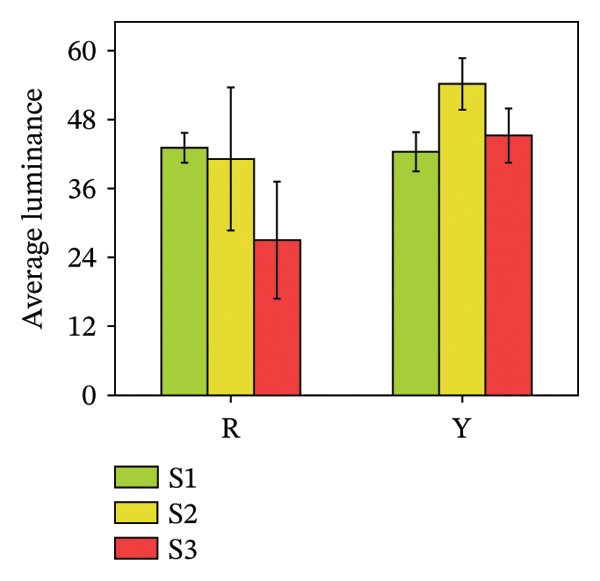
(a)

**Figure:**
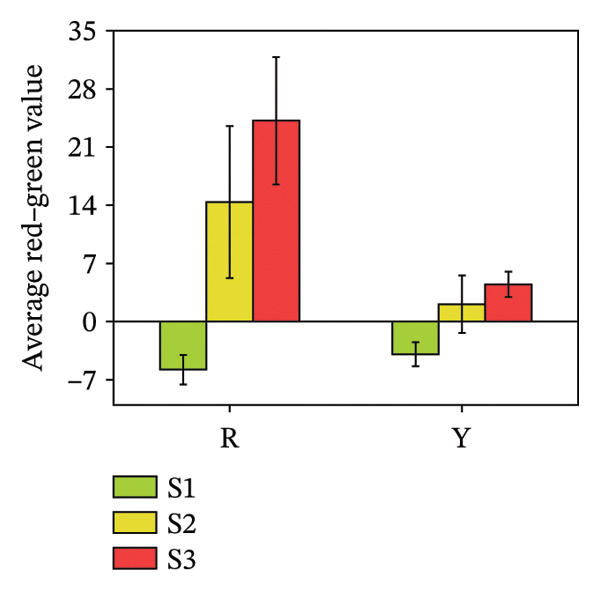
(b)

**Figure:**
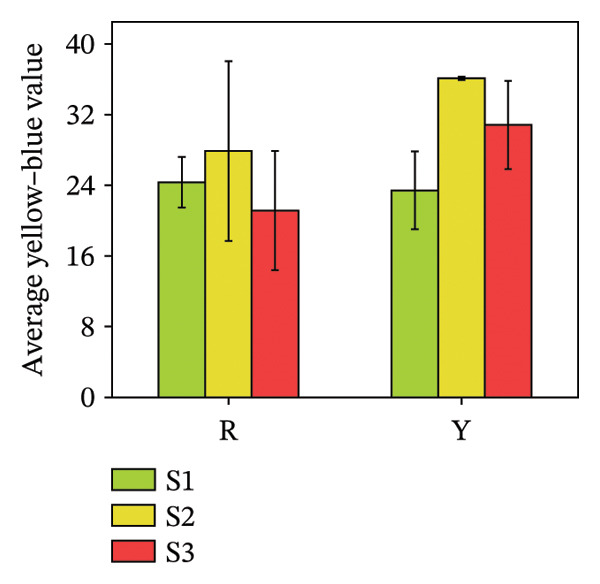
(c)

### 3.3. Nutritional Component Contents of *R. hirsutus* Fruits

#### 3.3.1. Sugar Contents of *R. hirsutus* Fruits

Sugars, as products of photosynthesis, are essential for both energy metabolism and the synthesis of structural and storage polysaccharides, such as cellulose and starch [29]. Glucose, fructose, sucrose, mannose, trehalose, maltose, etc., are sugars present in plants. Quantitative analysis revealed a distinct disparity in their individual concentrations (Figure 5). The glucose content in both colored fruits at S3 did not exceed 25 mg/g, significantly lower than the other two sugars (both R and Y exceeded 60 mg/g at S3). From S1 to S3, fructose content increased by 14.90% in R and 18.65% in Y; sucrose increased 3.82 times in R and 4.66 times in Y; whereas glucose increased 12.01 times in R and 15.49 times in Y. The increase in fructose was markedly smaller than that of glucose and sucrose. Statistical analysis revealed no significant difference in glucose concentration between the red‐ and yellow‐fruited varieties.

FIGURE 5Fructose (a), glucose (b), and sucrose contents (c) in the fructification of *R. hirsutus*. Note: Red (R) and yellow (Y) fruits of *R. hirsutus* are shown. Data are presented as mean ± SD (*n* = 3). Statistical significance was assessed using one‐way ANOVA followed by Fisher’s LSD post hoc test. Within the figure, identical letters assigned to values reflect a lack of statistical significance (*p* > 0.05), and distinct letters mark statistically significant differences (*p* < 0.05).

**Figure:**
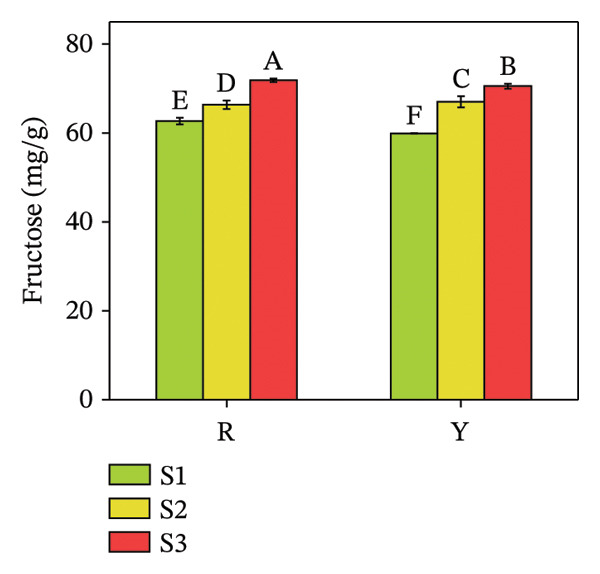
(a)

**Figure:**
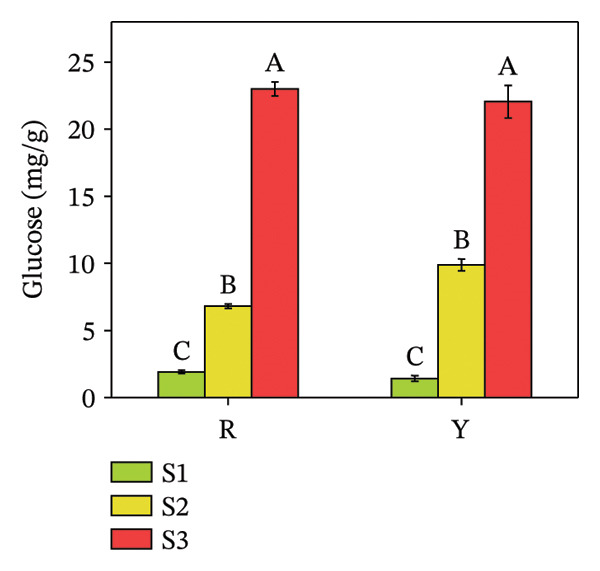
(b)

**Figure:**
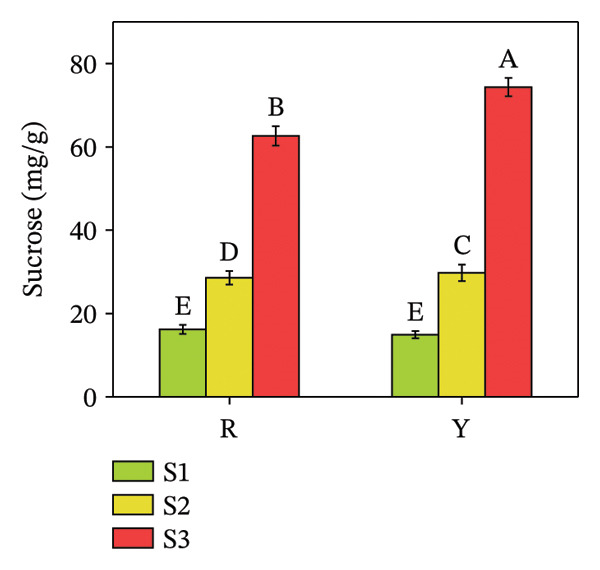
(c)

#### 3.3.2. TA and SSCs of *R. hirsutus* Fruits

TA was quantified by NaOH‐phenolphthalein titration [30], with results calculated as citric acid equivalents (factor: 0.064; *n* = 3). Figure 6 illustrates that TA levels diverged markedly between ripe fruits of different colors. Specifically, Y fruits exhibited a higher value (0.89%) than R fruits (0.73%). In contrast, SSC, predominantly composed of soluble sugars, showed no statistical difference between the red and yellow varieties.

FIGURE 6Titratable acidity (a) and soluble solid (b) content in the fructification of *R. hirsutus*. Note: R represents red fruits of *R. hirsutus*; Y represents yellow fruits of *R. hirsutus*. Titratable acidity is expressed as percentage (%, citric acid equivalent). Soluble solid content is expressed as °Brix.

**Figure:**
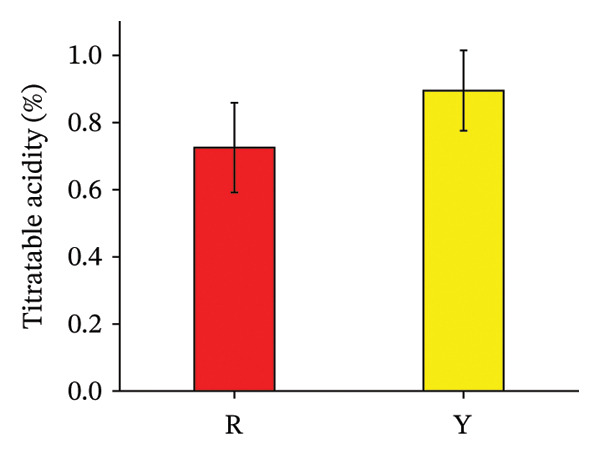
(a)

**Figure:**
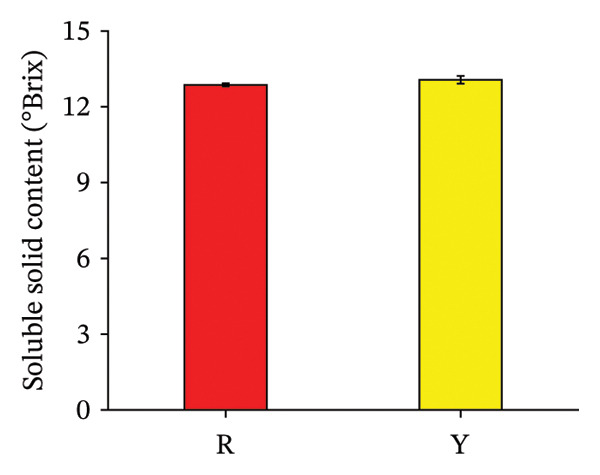
(b)

Solid–acid ratio (SAR) refers to the value obtained from SSC divided by TA [31]. According to Table 1, R and Y fruits exhibited SARs of 17.74 and 14.59, with average flavor scores of 8.75 and 8, respectively.

**TABLE 1:** Solid–acid ratio and flavor average value in the fructification of *R. hirsutus*.

Varieties	Solid–acid ratio	Flavor average value
Red fruit (R)	17.74	8.75
Yellow fruit (Y)	14.59	8

*Note:* Data are presented as mean ± SD (*n* = 6). Solid–acid ratio (SAR) was calculated as SSC/TA. Flavor scores are based on a 10‐point hedonic scale.

#### 3.3.3. Protein Content of *R. hirsutus* Fruits

**FIGURE 7:**
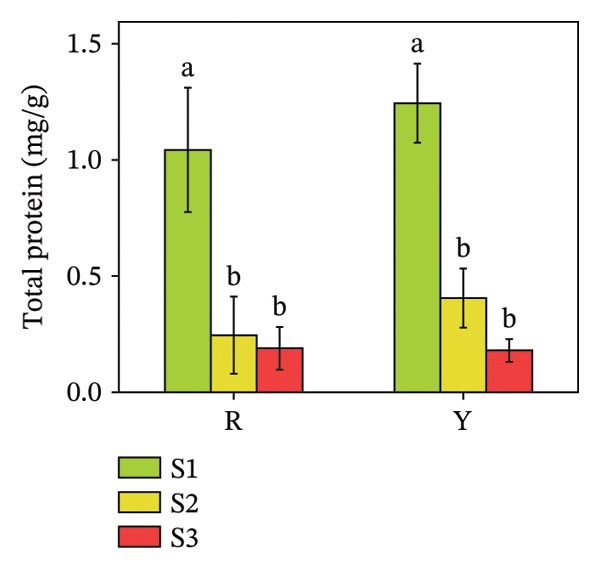
Total protein in the fructification of *R. hirsutus*. Note: Red (R) and yellow (Y) fruits of *R. hirsutus* are shown. Data are presented as mean ± SD (*n* = 3). Statistical significance was assessed using one‐way ANOVA followed by Fisher’s LSD post hoc test. Within the figure, identical letters assigned to values reflect a lack of statistical significance (*p* > 0.05), and distinct letters mark statistically significant differences (*p* < 0.05).

An analysis of the protein content was conducted to evaluate functional substance differences between the two colored fruit varieties. As presented in Figure 7, the protein content of both color types of *R. hirsutus* fruits reached the maximum at the S1 stage: 1.11 mg/g for R fruits and 1.32 mg/g for Y fruits. The protein content decreased with the growth process; the protein content at the S2 stage decreased significantly compared with that at the S1 stage, while the decrease in the protein content at the S3 stage compared with that at the S2 stage was not significant. The protein content showed no significant intervarietal differences when compared at the same developmental phase.

#### 3.3.4. Amino Acid Contents of *R. hirsutus* Fruits

According to Table 2, the total amino acid (TAA) content reached 1835.68 μg/g in R fruits, contrasted with a higher level of 2263.52 μg/g in Y fruits. In R fruits, the essential amino acid (EAA) fraction was 311.03 μg/g, comprising 16.94% of the total, while non–essential amino acids (NEAA), at 1524.65 μg/g, accounted for the remaining 83.06%. For the yellow fruits, EAA represented 17.97% (406.86 μg/g) of the total, while NEAA constituted 82.03% (1856.65 μg/g). The ideal protein criteria, as defined by the Food and Agriculture Organization/World Health Organization, recommend that both the EAA/TAA and EAA/NEAA ratios be above 40% and 60%, respectively [32]. Nevertheless, the EAA/TAA and EAA/NEAA ratios in R fruits were 16.94% and 20.39%, respectively, while those in Y fruits were 17.97% and 21.91%. Neither fruit type met the FAO/WHO standard values.

**TABLE 2:** Amino acid content in fructification of *R. hirsutus*.

Amino acid	R (*μ*g/g)	The proportion of total amino acids in R (%)	Y (*μ*g/g)	The proportion of total amino acids in Y (%)	Increase in Y relative to R amino acid (%)
Gln	2.69 ± 0.01s	0.15	2.8 ± 0ax	0.12	4.17
Tau	0.2 ± 0.01t	0.01	0.15 ± 0ba	0.01	−28.98
Hyp	0.41 ± 0.01t	0.02	0.53 ± 0az	0.02	30.55
Asn	305.38 ± 0.52b	16.64	243.69 ± 0.37ac	10.77	−20.20
Hcy	0.34 ± 0.01t	0.02	0.19 ± 0ba	0.01	−44.44
SAMe	19.66 ± 0op	1.07	25.58 ± 0.03ar	1.13	30.14
Hyd	0.07 ± 0t	0.004	0.07 ± 0ba	0.003	10.71
The	21.88 ± 0.29n	1.19	11.07 ± 0.02at	0.49	−49.42
Cit	2.91 ± 0.04rs	0.16	8.79 ± 0.01au	0.39	202.42
Trp	9.41 ± 0.13q	0.51	19.91 ± 0.02as	0.88	111.66
L‐Kyn	0.61 ± 0.01t	0.03	1.1 ± 0ay	0.05	79.90
SAH	0.99 ± 0.02t	0.05	1.09 ± 0ay	0.05	9.76
GABA	127.42 ± 1.59d	6.94	80.99 ± 0.02ai	3.58	−36.44
β‐Ala	34.53 ± 0.16l	1.88	28.83 ± 0.01ap	1.27	−16.51
Cys	0.11 ± 0.01t	0.01	0.11 ± 0ba	0.005	1.64
Leu	38.32 ± 0.59k	2.09	42.64 ± 0.01am	1.88	11.29
ILe	18.19 ± 0.3p	0.99	31.64 ± 0.05ao	1.40	73.97
Gly	4.46 ± 0.01r	0.24	4.52 ± 0.01av	0.20	1.36
Ala	402.86 ± 3.5a	21.95	564.14 ± 0.92aa	24.92	40.03
Ser	62.69 ± 2.22h	3.41	95.71 ± 0.16ag	4.23	52.68
Pro	66.85 ± 0.42g	3.64	173.04 ± 0.09ad	7.64	158.84
Val	39.05 ± 0.51k	2.13	53.86 ± 0.03aj	2.38	37.94
Thr	109.97 ± 0.82f	5.99	126.22 ± 0.07ae	5.58	14.78
Asp	125.52 ± 0.33e	6.84	116.03 ± 0.06af	5.13	−7.56
Lys	3.94 ± 0rs	0.21	4.06 ± 0aw	0.18	3.09
Glu	245.86 ± 1.81c	13.39	384.54 ± 0.43ab	16.99	56.41
Met	28.27 ± 0.18m	1.54	38.41 ± 0.04an	1.70	35.90
His	34.92 ± 0.66l	1.90	82.23 ± 0.09ah	3.63	135.49
Phe	57.24 ± 0.57i	3.12	46.29 ± 0.08al	2.05	−19.13
Arg	51.18 ± 0.42j	2.79	48.19 ± 0.08ak	2.13	−5.85
Tyr	19.76 ± 0.1o	1.08	27.06 ± 0.04aq	1.20	36.93
EAA	311.03	16.94	406.86	17.97	6.08
NEAA	1524.65	83.06	1856.65	82.03	−1.24
TAA	1835.68		2263.51		23.31

*Note:* Lowercase letters in the same column indicate significant differences in amino acid contents among different varieties (*p* < 0.05).

A comparative analysis revealed that statistically significant differences existed in the amino acid contents between the two tested samples. The contents of multiple amino acids in Y samples increased sharply compared with those in R samples, especially proline (Pro, +158.84%), histidine (His, +135.49%), citrulline (Cit, +202.42%), and tryptophan (Trp, +111.66%). In addition, the contents of isoleucine (Ile), L‐kynurenine (L‐Kyn), glutamic acid (Glu), and serine (Ser) also increased significantly by more than 50%. In contrast to the minor fluctuations observed for most amino acids, threonine (Thr) displayed a marked 49.42% reduction. Asparagine (Asn) was identified as the most abundant component in both fruit types, with proportions of 16.64% (305.38 μg/g) and 10.77% (243.69 μg/g) in the R and Y fruits, respectively. Hydroxylysine (Hyl), by comparison, registered the lowest quantities at 0.068 μg/g (R) and 0.075 μg/g (Y).

#### 3.3.5. Mineral Elements in *R. hirsutus* Fruits

Mineral elements play a critical role in supporting normal growth and development in plant and human systems. As the human body lacks the capacity to endogenously produce minerals, they must be acquired through dietary sources [33]. Therefore, the intake of mineral elements from plant fruits is one of the main ways for humans to obtain nutrients. Figure 8 demonstrates that the concentrations of 9 mineral elements diverged significantly between the two fruit color phenotypes of *R. hirsutus*. In terms of total mineral content, R fruits contained 3.32 g/kg macroelements and 29.09 mg/kg trace elements, versus 3.27 g/kg and 23.62 mg/kg in Y fruits. Regarding individual macroelements, potassium (K) was the most abundant (1.84 g/kg in R; 1.96 g/kg in Y) and sodium (Na) the least (0.15 g/kg in R; 0.11 g/kg in Y). The corresponding K/Na ratios stood at 12.27 for the R phenotype and 17.82 for the Y phenotype. Regarding trace elements, iron (Fe) had the highest content in R fruits (12.01 mg/kg), a value that was significantly 3.5 mg/kg higher than that in Y fruits. For Y fruits, zinc (Zn) exhibited the highest trace element content at 9.25 mg/kg. Selenium (Se) had the lowest content in both R and Y fruits, with concentrations of 0.05 mg/kg and 0.04 mg/kg, respectively. Collectively, the total trace element content in R fruits was higher than that in Y fruits.

FIGURE 8Contents of macroelements (a) and trace elements (b) in fructification of *R. hirsutus*. Note: Red (R) and yellow (Y) fruits of *R. hirsutus* are shown. Data are presented as mean ± SD (*n* = 3). Statistical significance was assessed using one‐way ANOVA followed by Fisher’s LSD post hoc test. Within the figure, identical letters assigned to values reflect a lack of statistical significance (*p* > 0.05), and distinct letters mark statistically significant differences (*p* < 0.05).

**Figure:**
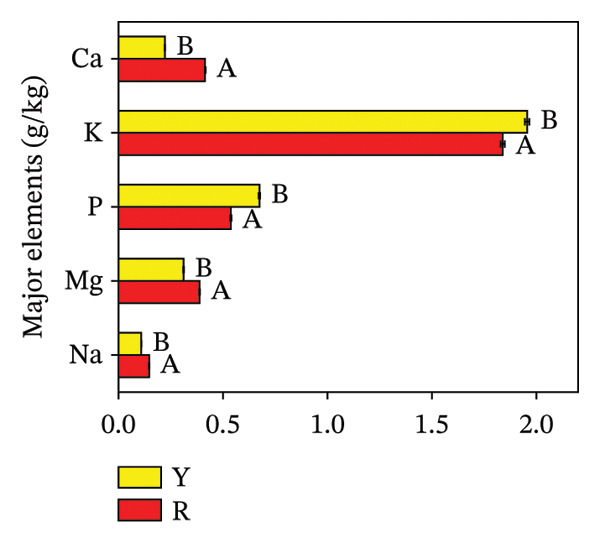
(a)

**Figure:**
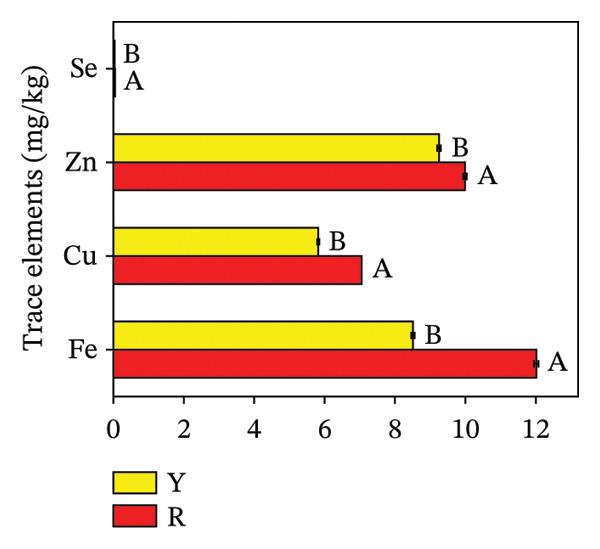
(b)

### 3.4. Antioxidant Substance Contents in *R. hirsutus* Fruits

#### 3.4.1. Total Phenol and Flavonoid Contents in *R. hirsutus* Fruits

Phenolic substances, specifically total phenols and flavonoids, represent major contributors to the antioxidant activity observed in plants [34]. Analyses of total phenols and flavonoids were therefore conducted. Figure 9 indicates a marked disparity in phenolic content favoring the R fruits, while flavonoid accumulation showed no significant intervarietal difference at the same stage. Specifically, S1 stage readings documented 2.85 mg/g total phenols and 2.17 mg/g flavonoids in R fruits, compared to 2.56 and 1.95 mg/g, respectively, in Y fruits. In line with this, both components underwent a remarkable reduction throughout the growth period.

FIGURE 9Contents of total phenols (a) and flavonoids (b) in the fructification of *R. hirsutus*. Note: Red (R) and yellow (Y) fruits of *R. hirsutus* are shown. Data are presented as mean ± SD (*n* = 3). Statistical significance was assessed using one‐way ANOVA followed by Fisher’s LSD post hoc test. Within the figure, identical letters assigned to values reflect a lack of statistical significance (*p* > 0.05), and distinct letters mark statistically significant differences (*p* < 0.05).

**Figure:**
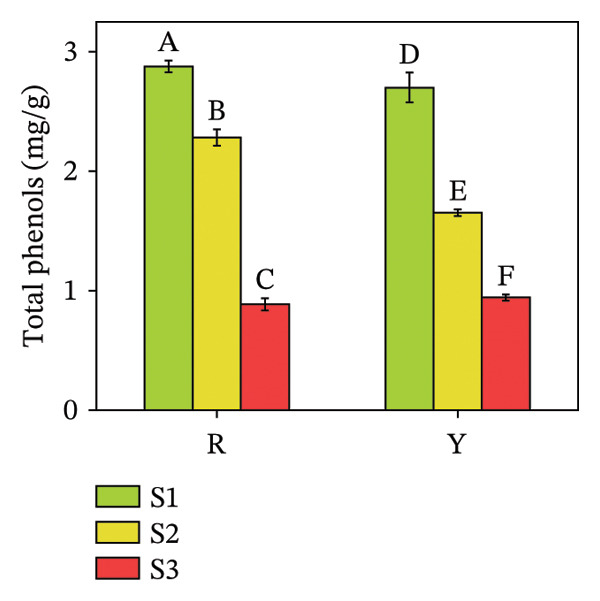
(a)

**Figure:**
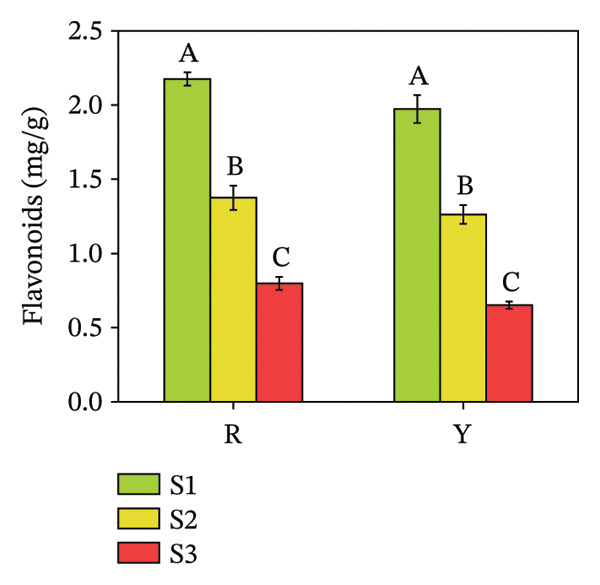
(b)

#### 3.4.2. Anthocyanin and GSH Contents in *R. hirsutus* Fruits

GSH is a low‐molecular‐weight nonenzymatic substance widely present in cells and participates in antioxidant metabolic reactions [35, 36]. Figure 10 illustrates that the anthocyanin content in R fruits at the S3 stage was 0.93 mg/g, a value significantly higher than that in Y fruits, specifically approximately 4.14‐fold greater than the anthocyanin content of Y fruits. Regarding GSH content, R fruits exhibited a significant increase as ripening progressed, whereas Y fruits showed a decreasing trend in GSH content during the same process. Although Y fruits had a higher GSH content than R fruits at the S1 stage, R fruits surpassed Y fruits in terms of GSH content from the S3 stage onward.

FIGURE 10Contents of anthocyanins (a) and GSH (b) in the fructification of *R. hirsutus*. Note: Red (R) and yellow (Y) fruits of *R. hirsutus* are shown. Data are presented as mean ± SD (*n* = 3). Statistical significance was assessed using one‐way ANOVA followed by Fisher’s LSD post hoc test. Within the figure, identical letters assigned to values reflect a lack of statistical significance (*p* > 0.05), and distinct letters mark statistically significant differences (*p* < 0.05).

**Figure:**
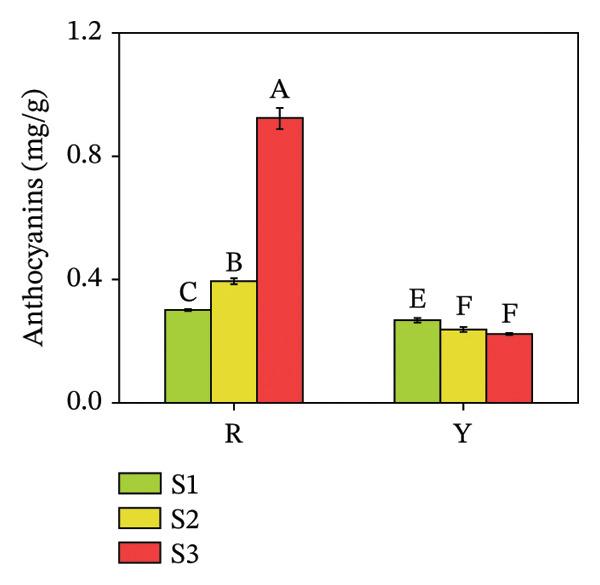
(a)

**Figure:**
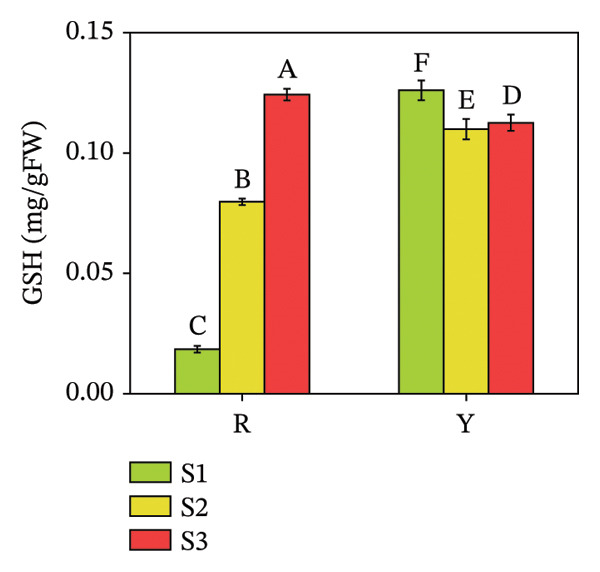
(b)

### 3.5. Activities of Antioxidant Enzymes (POD and SOD) in *R. hirsutus* Fruits

SOD is the first line of defense in the plant antioxidant system, and POD is an important enzyme in the plant antioxidant defense enzyme system [37]. A marked elevation in POD activity was observed in red fruits compared to yellow fruits throughout development. The POD activity of both color types of *R. hirsutus* fruits reached the maximum at the S2 stage (333.04 U/g for R fruits and 106.71 U/g for Y fruits), and the POD activity of R fruits was more than 3 times higher than that of Y fruits. SOD activity did not differ significantly between the two fruit types (Figure 11).

FIGURE 11Contents of POD (a) and SOD (b) in the fructification of *R. hirsutus*. Note: Red (R) and yellow (Y) fruits of *R. hirsutus* are shown. Data are presented as mean ± SD (*n* = 3). Statistical significance was assessed using one‐way ANOVA followed by Fisher’s LSD post hoc test. Within the figure, identical letters assigned to values reflect a lack of statistical significance (*p* > 0.05), and distinct letters mark statistically significant differences (*p* < 0.05).

**Figure:**
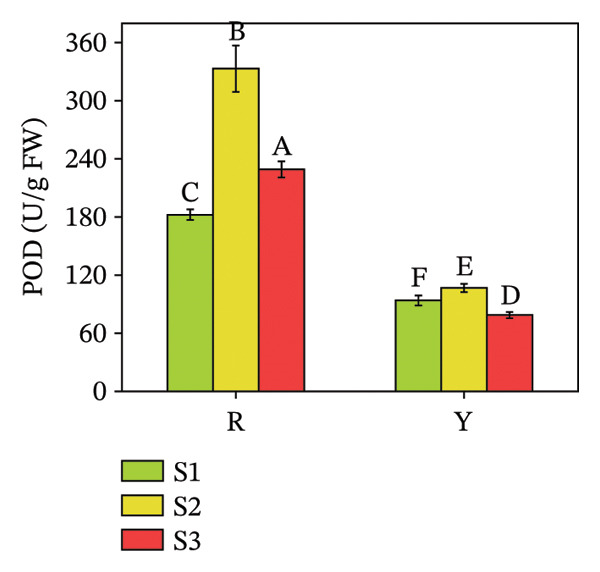
(a)

**Figure:**
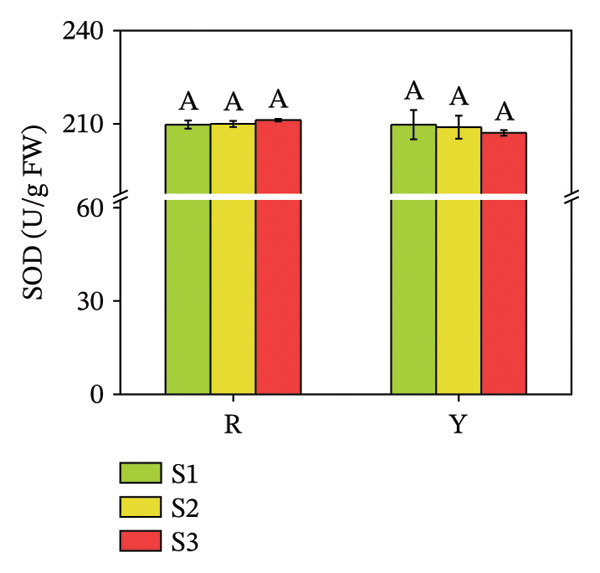
(b)

### 3.6. Correlation Analysis of *R. hirsutus* Fruit Indicators

To gain deeper insights into the correlations between the key biological indicators and fruit nutritional components of *R. hirsutus*, a correlation analysis was performed via calculation of the Pearson correlation coefficient. As presented in Figure 2, single fruit weight exhibited an extremely significant positive correlation with sucrose, with a correlation coefficient (*r*) of 0.99. Longitudinal diameter showed an extremely significant positive correlation with both single fruit weight and sucrose. Glucose and sucrose contents exhibited a strong positive correlation (*r* = 0.98). Transverse diameter displayed an extremely significant positive correlation with both longitudinal diameter and glucose, while flavonoids exhibited the same level of positive correlation with polyphenols; both pairs had a correlation coefficient of 0.97. Glucose also showed an extremely significant positive correlation with single fruit weight and fructose, with an *r* of 0.95. Furthermore, longitudinal diameter had a positive correlation with glucose and fructose, with correlation coefficients of 0.94 and 0.91, respectively. Fructose demonstrated a positive correlation with sucrose (*r* = 0.90), and protein showed a positive correlation with flavonoids (*r* = 0.90). In addition, fructose exhibited a positive correlation with transverse diameter and single fruit weight, with r values of 0.88 and 0.86, respectively. POD activity had a positive correlation with mineral elements (including total macroelements and total trace elements), with a correlation coefficient of 0.86. Finally, a significant positive correlation was observed between protein and polyphenol contents (*r* = 0.82).

Polyphenols exhibited an extremely significant negative correlation with glucose, with a correlation coefficient (*r*) of −0.98, while flavonoids showed an equally strong extremely significant negative correlation with glucose (*r* = −0.95). Flavonoids displayed an extremely significant negative correlation with fructose, and polyphenols had the same level of negative correlation with transverse diameter and sucrose; all these pairs shared a correlation coefficient of −0.94. Protein demonstrated an extremely significant negative correlation with fructose, and flavonoids exhibited this degree of negative correlation with both transverse diameter, longitudinal diameter and sucrose, with an r value of −0.92 for each. Flavonoids also showed an extremely significant negative correlation with single fruit weight, and polyphenols displayed the same correlation with longitudinal diameter and single fruit weight, with a correlation coefficient of −0.91 for these combinations. POD activity had a significant negative correlation with TAA content (*r* = −0.86). Finally, protein exhibited a negative correlation with longitudinal diameter and glucose, with respective correlation coefficients of −0.80.

## 4. Discussion and Conclusions

This study presents the first comprehensive evidence that fruit color variation in *R*. *hirsutus* is associated with extensive and previously uncharacterized differences in biochemical composition, antioxidant capacity, and nutritional value. By integrating analyses of morphology, sensory traits, sugars, organic acids, amino acids, minerals, phenolic compounds, and multiple antioxidant parameters across three developmental stages, our work extends significantly beyond previous studies that focused primarily on fruit size or ripening stages in red‐fruited *R. hirsutus*. The novel findings reported here, particularly the distinct amino acid profiles, sugar–acid balance, and antioxidant system dynamics of the yellow phenotype, offer new insights into the metabolic basis of color differentiation and its potential implications for nutrition, breeding, and functional food development. Below, we discuss these multidimensional differences in relation to fruit development, sensory quality, functional properties, and nutritional value.

1. Dynamic relationship between fruit morphological development and substance accumulation
 Fruit morphology (transverse diameter, longitudinal diameter, and single fruit weight) was highly significantly positively correlated with soluble sugar content (*p* < 0.01) [38], indicating synchronous sugar accumulation with fruit expansion during maturation. Analysis revealed a marked inverse correlation between protein and soluble sugar contents (*p* < 0.01). Flavonoid and total phenol contents were also significantly negatively correlated with fruit morphological indices and soluble sugar content (*p* < 0.05). This seesaw pattern reflects a stage‐specific shift in metabolic priorities during fruit development [39]: During S1, nitrogen metabolism is more vigorous, with active amino acid and protein synthesis for building cell structures and enzyme systems; upon entering S3, photosynthetic products are channeled more toward carbon metabolism pathways for sugar and starch synthesis, while nitrogen metabolism becomes relatively weaker [40], i.e., a transition from early growth (protein synthesis and secondary metabolite accumulation) to sugar accumulation and quality formation during ripening.
2. Relationship between Color Phenotype and Apparent Characteristics
 Statistically significant differences were observed in sensory attributes between *R. hirsutus* fruits of different color phenotypes. Y fruits had higher lightness (*L* ^∗^) and yellow–blue value (*b* ^∗^), indicating lighter and brighter color. R fruits darkened with ripening (increase in *a* ^∗^ value). The average red–green value (*a* ^∗^ = 14.39 ± 9.14) of R fruits at the S3 stage was significantly higher than that of Y fruits (*a* ^∗^ = 4.49 ± 1.52). The *a* ^∗^ value of R fruits exhibited a large coefficient of variation, which suggests that the ripeness of fruits within the same batch was inconsistent—with some in the color‐turning stage and others in the fully mature stage. In contrast, the coefficient of variation for the *a* ^∗^ value of Y fruits was small; this indicates that monitoring changes in the *a* ^∗^ value can effectively determine the optimal harvest time for Y fruits throughout the “immature ⟶ color‐turning ⟶ fully mature” developmental process.
3. Characteristics of Sugar–Acid Metabolism and Flavor Formation
 Flavor quality primarily depends on the composition, content, and ratio of soluble sugars (e.g., fructose, glucose, and sucrose) and organic acids [41]. *R. hirsutus* fruits are rich in fructose, glucose, and sucrose, constituting their main soluble solids [31]. The accumulation of these sugars is closely related to leaf photosynthetic intensity [42]. Significant differences existed in the sugar–acid composition between R and Y: *Fructose*: R was significantly higher than Y at S1 and S3 but significantly lower at S2. *Glucose*: Statistical analysis showed a lack of significant difference between the R and Y fruits. *TA*: The TA in Y fruits significantly surpassed that of the R fruits. Although the total soluble sugar content was relatively lower in R, its lower TA resulted in a significantly higher SAR and overall flavor score than in Y. This suggests better palatability (sweet–sour balance) for R fruits. Differences in sugar–acid content and ratio may stem from their varying energy demands and metabolic characteristics at different developmental stages.
4. Functional Substance Content and Physiological Significance
 Phenolic compounds, flavonoids, and proteins are well‐documented for their roles in plant antioxidant defense and stress resistance [43, 44]. *Protein*: The protein content in both fruit types was significantly higher at S1 than at S2 and S3 (no significant difference between the latter two), consistent with the high demand for protein during rapid growth in the green stage and reduced demand during ripening [45]. These differences in functional substance content suggest that red and yellow *R. hirsutus* fruits employ distinct metabolic strategies, though the underlying mechanisms require further investigation.
5. Differences in Antioxidant System Activity
 Key antioxidant indicators differed significantly between R and Y. Anthocyanin content in R fruits at maturity was substantially higher than in Y fruits, contributing to its red coloration and potentially enhancing antioxidant capacity [46]. GSH content increased during ripening in R fruits but decreased in Y fruits, resulting in higher GSH levels in R at maturity. POD activity was consistently higher in R fruits throughout development, peaking at the color‐turning stage at levels approximately threefold those of Y fruits. In contrast, SOD activity did not differ significantly between the two types. The enhanced antioxidant system in R fruits at maturity suggests a greater capacity to cope with oxidative stress [46, 47], which may contribute to cellular maintenance during ripening. Additionally, these compounds have documented health benefits in humans [8, 10, 13].
6. Mineral Element Composition and Significance
 The content of all 9 measured mineral elements differed significantly between R and Y fruits. *Potassium* (K) was the most abundant microelement and plays vital roles in maintaining cell turgor, regulating stomata, and promoting photosynthesis. The characteristic of high K and low Na is beneficial for maintaining fluid balance and cardiovascular health [47–49]. Iron (Fe): The Fe content in R fruits was 12.01 mg/kg, which was significantly higher than that in Y fruits. As a key element for chlorophyll synthesis and photosynthesis/respiration [50], higher Fe content may support enhanced photosynthetic and respiratory efficiency in R fruits. *R. hirsutus* fruits are a good source of mineral elements, and their content differences are significant for both plant growth and development and as a source of human nutritional supplementation.
7. Amino Acid Composition and Nutritional Value
 Amino acids are fundamental for protein synthesis and are crucial for the nutritional value of fruits [51, 52]. There were significant differences in amino acid composition between the two *R. hirsutus* fruit types. Specifically, for TAAs: The TAA content of Y fruits was 2263.52 μg/g, which was significantly higher than the 1835.68 μg/g measured in R fruits. In terms of EAA: Y fruits contained 406.86 μg/g of EAA (accounting for 17.97% of TAA), a value significantly higher than that of R fruits (311.03 μg/g, representing 16.94% of TAA). Regarding NEAA: The proportions of NEAA relative to TAA were relatively similar between the two, with Y fruits at 82.03% and R fruits at 83.06%. Although the EAA proportions of both did not fully meet the FAO/WHO ideal protein standards, they both provide a considerable amount of amino acids, with Y having an advantage in total and EAA content.

## 5. Summary and Prospects

**FIGURE 12:**
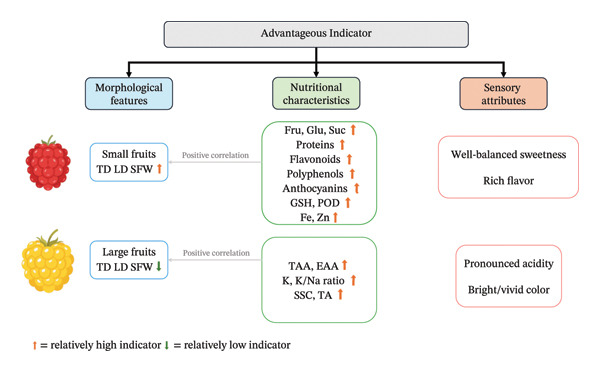
Comparison of superior traits of *R. hirsutus*.

This study provided a comprehensive analysis of significant differences between red and yellow *R. hirsutus* fruits across four key dimensions: morphological traits, physicochemical properties, bioactive compounds, and antioxidant activity. For red fruits (R): At the fully mature stage, they exhibited a deep red color, which was associated with a high *a* ^∗^ value and elevated anthocyanin content. In terms of eating quality, R fruits had superior palatability, characterized by a higher SAR and flavor score. They also demonstrated robust antioxidant capacity, supported by higher contents of total phenols and GSH, as well as increased POD activity. Additionally, R fruits had a higher iron (Fe) content. For yellow fruits (Y): They displayed a vibrant appearance, reflected by higher *L* ^∗^ and *b* ^∗^ values. Nutritionally, Y fruits contained higher levels of TAAs and EAAs, along with elevated TA. Furthermore, Y fruits had a greater content of most mineral elements compared to R fruits. These differences not only reflect the regulatory mechanisms of fruit development metabolism [38, 39] but also provide an important basis for cultivar selection and cultivation management: Consumers preferring sweet–sour balanced taste might choose red fruits, while those focusing on amino acid nutrition or preferring higher acidity might choose yellow fruits. Both fruit types are rich in bioactive compounds, possessing high edible and potential medicinal value. Notably, research on yellow‐fruited *R. hirsutus* is relatively scarce, leaving broad research space regarding its unique metabolic pathways, environmental adaptability, and utilization value (Figure 12).

## Author Contributions

Xiaobao Tong: validation, data curation, formal analysis, investigation, writing original draft, writing–review and editing, and visualization. Xingru Wei: investigation, writing original draft, writing–review and editing, and visualization. Xinyue Ping and Liangqin Liu: data analysis, supervision, and resources. Qilong Zeng and Yanqin Jiang: writing–review and editing. Xiaomin Wang: conceptualization, investigation, writing–review and editing, supervision, project administration, and funding acquisition.

## Funding

Open access funding was provided by the Jiangsu Key Laboratory for Conservation and Utilization of Plant Resources (JSPKLB202411).

## Conflicts of Interest

The authors declare no conflicts of interest.

## Data Availability

The dataset supporting the conclusions of this article is included in this article.
